# Supplementary material for: Content-rich biological network constructed by mining PubMed abstracts
Source: BMC Bioinformatics. 2004 Oct 8;5:147. doi: 10.1186/1471-2105-5-147 (PMC528731; doi:10.1186/1471-2105-5-147)
Supplement: Additional File 5 — The original Chilibot query results of the term "long-term potentiation (LTP)" and 22 other terms, limiting the latest references analyzed to the years 1990, 1995, 2000, and 2004. [file 1471-2105-5-147-S5.bz2 › chilibotAdditionalFile5/ltp2004/html/PLC_ACTIN.html]

 


 **PLC** and **ACTIN** 
  
Found 127 abstracts in PubMed,  **30 abstracts were retrieved and analyzed**.  


---

 Search Google  |
 PDF files only 
|  EDU domain only 

---

**Interactive relationship** (e.g. stimulation, inhibition, etc)

- The potencies to cause disorganization of  **actin**  filaments were consistent with the potencies of these agents as inhibitors of  **PLC**  beta and enhancers of tight junction permeability.  Ref: 12538823 J Pharmacol Exp Ther, 2003
- These results provide strong evidence that  **PLC**  beta inhibition leads to increased tight junction permeability across MDCK cell monolayers through disorganization of  **actin**  filaments.  Ref: 12538823 J Pharmacol Exp Ther, 2003
- B Relative to WT cells exposed to oxidant, monolayers of transfected cells over expressing  **PLC**  gamma 2.3 fold were protected as indicated by decreases in all measures of iNOS driven pathway and enhanced  **actin**  and barrier integrity.  Ref: 12788694 Am J Physiol Cell Physiol< MedlineTA>Am J Physiol Cell Physiol, 2003
- These results further show that  **PLC**  beta modulates tight junction permeability by affecting  **actin**  filament organization.  Ref: 12538823 J Pharmacol Exp Ther, 2003
- Removing GPI Ps glycosylphosphatidylinositol anchored proteins from the cell surface by PI  **PLC**  phophatidylinositol phospholipase C or disrupting the cell  **actin**  cytoskeleton by CD cytochalasin D reduced VacA induced vacuolation of cells Ricci et al.  Ref: 14676190 J Biol ChemJ Biol Chem, 2003
- To conclude, our findings suggest that  **actin**  may play a pivotal role in the response of OK cells to TNF alpha and implicate Cdc42 in directly regulating  **PLC**  gamma1 activity.  Ref: 14699068 Mol Biol CellMol Biol Cell, 2003
- During capacitation, there is also an increase in protein tyrosine phosphorylation dependent  **actin**  polymerization and in the membrane bound phospholipase C  **PLC** .  Ref: 12887084 Cell Mol Biol (Noisy-le-grand), 2003

**Parallel relationship** (e.g. studied together, co-existance, homology, etc.)

- Staurosporine treatment induced relocation of tyrosine phosphorylated phospholipase C gamma1  **PLC**  gamma1 to the tips of lamellipodia where  **actin**  assembly was initiated.  Ref: 12890758 J Cell Sci, 2003
- TNF alpha promotes survival of OK cells via Cdc42 induced  **PLC**  gamma 1 activation and  **actin**  filament redistribution.  Ref: 14699068 Mol Biol CellMol Biol Cell, 2003
- Conclusions 1 EGF protects against oxidative stress disruption of the intestinal barrier integrity by stabilizing the F  **Actin** , in large part, through the activation of  **PLC**  gamma and down regulation of iNOS pathway.  Ref: 12788694 Am J Physiol Cell Physiol< MedlineTA>Am J Physiol Cell Physiol, 2003
- Staurosporine induced recruitment of filamentous  **actin**  F  **actin** , cortactin, filamin, Arp2 3 complex, Rac1 GTPase and phospholipase C gamma1  **PLC**  gamma1 to lamellipodia.  Ref: 14729058 Exp Cell Res, 2004
- The inactive Cdc42 T17N and the  **PLC**  gamma1 inhibitor U73122 prevented  **actin**  redistribution and depolymerization confirming that both signaling molecules are responsible for the reorganization of  **actin** .  Ref: 14699068 Mol Biol CellMol Biol Cell, 2004
- Inhibition of Oxidant Induced NF kappa B Activation I kappa B alpha Degradation and Instability of F  **actin**  Cytoskeletal Dynamics Barrier Function by EGF Key Role of  **PLC**  gamma Isoform.  Ref: 14724221 J Pharmacol Exp TherJ Pharmacol Exp Ther, 2004
- Furthermore, ATP, an activator of  **PLC**  beta, attenuated U73122 induced increase in tight junction permeability as well as disorganization of  **actin**  filaments.  Ref: 12538823 J Pharmacol Exp Ther, 2003
- Inhibition of Oxidant Induced iNOS Dependent Nitration and Disassembly of F  **actin**  Cytoskeleton and Loss of Epithelial Barrier Function by EGF Role of  **PLC**  gamma Isoform.  Ref: 12788694 Am J Physiol Cell Physiol< MedlineTA>Am J Physiol Cell Physiol, 2003
